# KLK5 and KLK7 Ablation Fully Rescues Lethality of Netherton Syndrome-Like Phenotype

**DOI:** 10.1371/journal.pgen.1006566

**Published:** 2017-01-17

**Authors:** Petr Kasparek, Zuzana Ileninova, Olga Zbodakova, Ivan Kanchev, Oldrich Benada, Karel Chalupsky, Maria Brattsand, Inken M. Beck, Radislav Sedlacek

**Affiliations:** 1 Laboratory of Transgenic Models of Diseases, Division BIOCEV, Institute of Molecular Genetics of the Czech Academy of Sciences, v.v.i., Vestec, Czech Republic; 2 Faculty of Sciences, Charles University in Prague, Prague, Czech Republic; 3 Czech Centre for Phenogenomics, Division BIOCEV, Institute of Molecular Genetics of the Czech Academy of Sciences, v.v.i., Vestec, Czech Republic; 4 Laboratory of Molecular Structure Characterization, Institute of Microbiology of the Czech Academy of Sciences, v.v.i., Prague, Czech Republic; 5 Department of Medical Biosciences, Pathology, Umea University, Umea, Sweden; Stanford University School of Medicine, UNITED STATES

## Abstract

Netherton syndrome (NS) is a severe skin disease caused by the loss of protease inhibitor LEKTI, which leads to the dysregulation of epidermal proteases and severe skin-barrier defects. KLK5 was proposed as a major protease in NS pathology, however its inactivation is not sufficient to rescue the lethal phenotype of LEKTI-deficient mice. In this study, we further elucidated the *in vivo* roles of the epidermal proteases in NS using a set of mouse models individually or simultaneously deficient for KLK5 and KLK7 on the genetic background of a novel NS-mouse model. We show that although the ablation of KLK5 or KLK7 is not sufficient to rescue the lethal effect of LEKTI-deficiency simultaneous deficiency of both KLKs completely rescues the epidermal barrier and the postnatal lethality allowing mice to reach adulthood with fully functional skin and normal hair growth. We report that not only KLK5 but also KLK7 plays an important role in the inflammation and defective differentiation in NS and KLK7 activity is not solely dependent on activation by KLK5. Altogether, these findings show that unregulated activities of KLK5 and KLK7 are responsible for NS development and both proteases should become targets for NS therapy.

## Author Summary

Netherton syndrome (NS) is a genetic skin disorder caused by the loss of protease inhibitor LEKTI, which leads to the dysregulation of epidermal proteases and severe skin-barrier defects. In this work, we aimed to explore the molecular mechanisms underlying this disease using a novel mutant mouse model for NS, which is based on mimicking a causative mutation known from human patients. This novel model reproduces the symptoms of NS and thus provides a useful tool to study the NS pathology in a complex *in vivo* environment. Most importantly, by combination of this NS-mouse model with mutant mice individually or simultaneously deficient for proteases KLK5 and KLK7, we elucidated the complex proteolytic networks that are dysregulated in the absence of LEKTI. We show that although the single ablation of KLK5 or KLK7 is not sufficient to rescue the lethal effect of LEKTI-deficiency, simultaneous deficiency of both KLKs completely rescues the epidermal barrier and the postnatal lethality. Our results also provide novel insights into the roles of KLK5 and KLK7 in the inflammation and differentiation defects that are associated with NS. Based on these findings, we propose that both, KLK5 and KLK7 should become targets for NS therapy.

## Introduction

Netherton syndrome (NS) is a life-threatening autosomal recessive disorder that affects approximately one in 200 000 newborn children [1,2]. Newborns suffering from NS exhibit congenital *ichthyosiform erythroderma* with scaly and peeling skin, resulting in severe disruption of epidermal barrier, which in some cases is fatal. These conditions may improve with age and older patients often show less severe *ichthyosis* exhibiting erythematous plaques with double-edged scales at the periphery [1–3]. The hair of NS patients is usually thin, fragile and the patients often develops “bamboo hair”, a hair shaft defect where the distal part of the hair shaft is invaginated into its proximal part [4]. NS may be also associated with growth retardation, asthma, food allergies, and elevated serum levels of IgE [1,5].

NS is caused by mutations in *SPINK5* gene (serine protease inhibitor Kazal-type 5) that encodes LEKTI (lympho-epithelial Kazal-type related inhibitor), an inhibitor of serine proteases expressed in the epidermis and other stratified epithelia [6]. Full length LEKTI consists of 15 inhibitory domains (D1—D15) and upon synthesis undergoes proteolytic processing into multiple bioactive fragments containing one to six domains with distinct inhibitory specificities [7,8]. LEKTI has been reported to inhibit several proteases including plasmin, trypsin, subtilisin A, cathepsin G, elastase, caspase-14 [9–11] as well as members of the family of kallikrein-related peptidases (KLK), mainly KLK5, KLK7 and KLK14 [12–14]. Unregulated activity of KLK5 and possibly also KLK7 is considered a major source of pathology in NS. Spink5 deficient mice show increased proteolytic activities of KLK5 and KLK7 [15], which corresponds to elevated tryptic and chymotryptic activities described in NS patients [16,17]. KLK5 also initiates a proteolytic cascade by proteolytic activation of KLK7 and KLK14, that leads to degradation of corneodesmosomal proteins desmoglein1 (DSG1), desmocollin1 (DSC1), and corneodesmosin (CDSN)[18]. Premature degradation of corneodesmosomes results in detachment of the *stratum corneum* (SC) and disruption of the epidermal barrier in NS patients [17]. Upregulated proteolytic activity can further contribute to skin barrier defects by abnormal processing of profilaggrin, a precursor protein which is proteolytically converted into physiologically active filaggrin monomers. Filaggrin is one of key players in maintaining skin hydratation and water retention of the epidermis [19]. Recently, KLK5 was shown to promote profilaggrin processing either via proteolytic activation of elastase 2, which cleaves filaggrin precursor proteins [20] or by direct degradation of profilaggrin [21]. In addition, previous studies using mouse models and human NS patients suggest that the unregulated activity of KLK5 contributes to the inflammatory response in the LEKTI-deficient epidermis by activation of protease-activated receptor 2 (PAR2)[22–24].

In this study, we revealed the *in vivo* roles of KLK5 and KLK7 using a set of mouse models that are simultaneously deficient for KLK5 and KLK7 on the genetic background of Netherton syndrome-like mouse model based on a mutation found in human patients. The close proximity of these genes (on the same locus) has so far prevented the generation of suitable animal models and therefore the *in vivo* roles of KLK5 and KLK7 could not be studied concurrently. Our study shows that individual functional ablation of KLK5 or KLK7 is not sufficient to rescue the lethal effect of *Spink5* mutation. In contrast, simultaneous deficiency of both KLK5 and KLK7 completely rescues the lethality allowing adult mice to survive to adulthood with a fully functional skin barrier.

## Results

### A135X mutation in LEKTI causes severe skin phenotype that leads to early postnatal lethality

**Fig 1:**
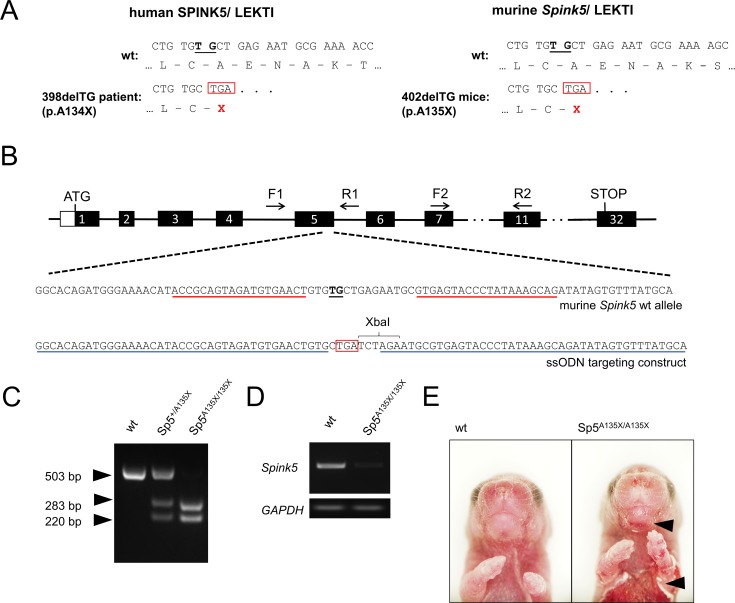
Generation of *Spink5* mutant mice. **(A)** Nucleotide and amino acid sequences of human SPINK5/LEKTI (left). The two-bp deletion 398delTG in exon5 of human SPINK5 gene results in a frame-shift and PTC (red box) as described in Raghunath et al., 2004(23). Comparison of corresponding sequences in murine Spink5/LEKTI (right) where deletion of TG nucleotides at position 402 (black underline) has the same impact as in humans. **(B)** Position of critical TG nucleotides (black underline) in the exon5 of murine Spink5 gene. TALEN-binding sites are marked with red underline, position of primers for PCR screening (F1, R1, F2, R2) are denoted. As a targeting construct, a single-stranded oligonucleotide having sequences homologous to wt DNA (blue underline) flanked desired mutation. Premature STOP codon (red box) and introduced new *Xba*I recognition sequence are depicted. **(C)** RFLP analysis of targeted mice. PCR product amplified from genomic DNA using primers F1 and R1 was digested using *Xba*I enzyme. Cleavage products of 283 and 220 bp originate from the positively targeted allele, a 503 bp fragment marks the wt allele. **(D)** Expression of Spink5 mRNA was analysed by semi-qPCR analysis in Sp5^A135X/A135X^ mice using primers F2 and R2. Expression of GAPDH was used as a control. **(E)** Phenotype of Sp5^A135X/A135X^ newborn pups. Areas of peeling skin are marked with black arrowheads.

To study NS pathology *in vivo*, we generated a new mouse model mimicking a causative mutation of *SPINK5* gene (398delTG; p.A134X) previously described in human patients [25]. Due to the similarity between the human and murine *SPINK5* nucleotide sequences, deletion of TG nucleotides at positions 402 and 403 of murine *Spink5* (402delTG) produces the premature termination codon (PTC) at a similar position as described in human patients (p.A135X) (Fig 1A). To introduce the mutation into mouse genomic DNA, we prepared TALE nucleases (TALENs) specific for the critical region of *Spink5* in combination with a single stranded oligonucleotide (ssODN) carrying the desired mutation (Fig 1B). Founders were screened for targeted incorporation of ssODN by RFLP analysis using *Xba*I restriction site as a marker (Fig 1C). Heterozygous mice carrying A135X mutation (hereafter referred to as Sp5^+/A135X^) did not show any obvious phenotype and were used to obtain Sp5^A135X/A135X^ mice, which showed dramatic downregulation of *Spink5*-RNA expression (Fig 1D). The presence of PTC in the *Spink5* transcript was confirmed by sequencing (S1 Fig). Sp5^A135X/A135X^ mice were born in normal Mendelian ratios, however they exhibited severe skin phenotype with exfoliating epidermis, predominantly localized in the abdominal and facial area (Fig 1E) and died within 12 hours after delivery. These phenotypical features mimic NS characteristics and correspond to previously published mouse models of Netherton syndrome [15,26–28].

### Inactivation of KLK5 and KLK7 on the genetic background of Sp5^A135X/A135X^ mice

**Fig 2:**
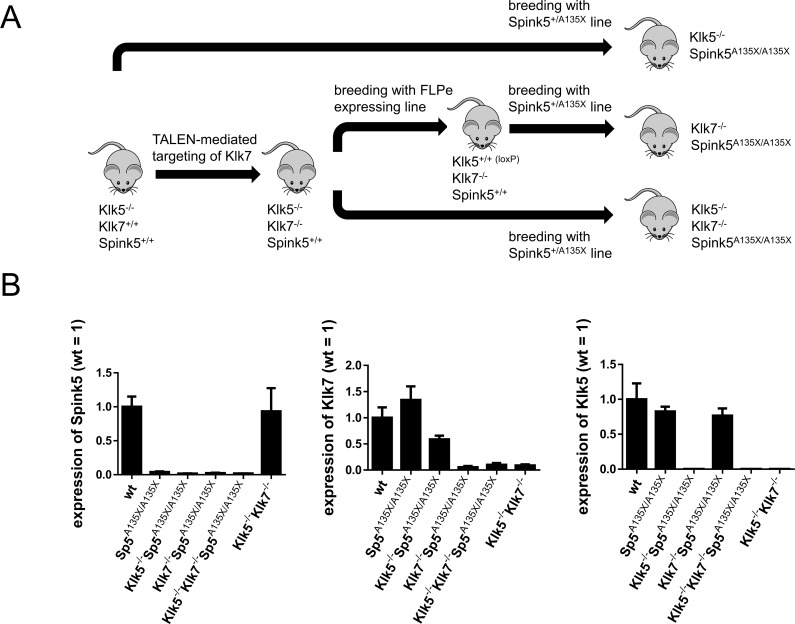
Generation of Spink5/Klk5/Klk7 mutant lines. **(A)** Klk5^-/-^ line (depicted as Klk5^-/-^Klk7^+/+^Spink5^+/+^), was used for preparation of Klk5^-/-^Klk7^-/-^ mice (depicted as Klk5^-/-^Klk7^-/-^Spink5^+/+^) by TALEN mutagenesis. Obtained Klk5^-/-^Klk7^-/-^ mice were further crossed to a Flippase (FLPe) expressing mouse line to allow conditionally expressed Klk5, thus generating Klk7^-/-^ mice (depicted as Klk5^+/+^(loxP)Klk7^-/-^Spink5^+/+^). Klk5^-/-^, Klk7^-/-^ and Klk5^-/-^Klk7^-/-^ lines were subsequently crossed with Spink5^+/-^ line to obtain Klk5^-/-^Sp5^A135X/A135X^, Klk7^-/-^Sp5^A135X/A135X^, and Klk5^-/-^Klk7^-/-^Sp5^A135X/A135X^, i.e. double and triple KO lines. **(B)** Expression of Klk5, Klk7 and Spink5 at the mRNA level was quantified using qRT-PCR, n≥4 for each genotype, error bars represent standard deviations from mean.

To elucidate the roles of KLK5 and KLK7 in NS, we generated a set of KLK mutants (Klk5^-/-^, Klk7^-/-^, Klk5^-/-^Klk7^-/-^), which were crossed with Spink5^+/A135X^ line (Fig 2A). Klk5^-/-^ mice were generated by substitution of exon2 of *Klk5* gene with a tm1a-type targeting vector [29] (S2A Fig). As *Klk5* and *Klk7* are located within close proximity on mouse chromosome 7, generation of *Klk5* and *Klk7* double-deficient mouse by cross-breeding of individual KO lines is not possible. We therefore applied TALEN-mediated mutagenesis to disrupt the *Klk7* gene directly on the genetic background of Klk5^-/-^ mice by introduction of a frame-shift mutation in exon3 of *Klk7* gene (S3A Fig). Positively targeted mice were analysed by sequencing of genomic DNA and founder Klk7ex3-A containing 20 bp deletion in *Klk7* coding sequence (S3B Fig) was used to establish Klk5^-/-^Klk7^-/-^ double-deficient line. Targeting of *Klk7* was confirmed by cDNA sequencing (S3C Fig) and ablation of KLK7 protein was verified by western blot analysis (S3D Fig). Klk5^-/-^Klk7^-/-^ mice were further crossed to FLPe expressing mouse line to remove Klk5 KO cassette and re-constitute expression of *Klk5* (S2B Fig), thus generating Klk7^-/-^ mutant line (Fig 2A). Klk5^-/-^, Klk7^-/-^ and Klk5^-/-^Klk7^-/-^ newborn P0 mice were phenotypically indistinguishable from their control littermates and did not shown any obvious cutaneous phenotype. KLK-deficient mutants were further bred to Sp5^+/A135X^ line in order to generate double Klk5^-/-^Sp5^A135X/A135X^, Klk7^-/-^Sp5^A135X/A135X^, and triple Klk5^-/-^Klk7^-/-^Sp5^A135X/A135X^ mutant mice (Fig 2A). Analysis of mRNA levels confirmed the loss of the targeted gene’s expression (Fig 2B).

### Simultaneous inactivation of KLK5 and KLK7 rescues the lethal phenotype of Sp5^A135X/A135X^ mice

**Fig 3:**
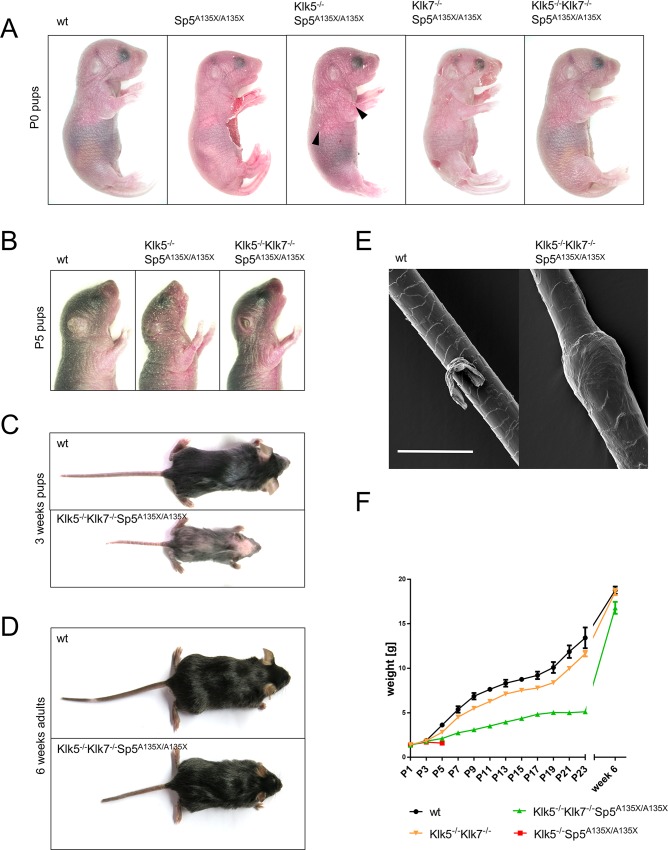
Gross phenotype of Spink5- Klk- deficient mutant lines. **(A)** Phenotype of wt, Sp5^A135X/A135X^, Klk5^-/-^Sp5^A135X/A135X^, Klk7^-/-^Sp5^A135X/A135X^ and Klk5^-/-^Klk7^-/-^Sp5^A135X/A135X^ mice 12 hours after birth. Peeling skin was observed in Sp5^A135X/A135X^ and Klk7^-/-^Sp5^A135X/A135X^ and to lesser extent in Klk5^-/-^Sp5^A135X/A135X^ pups (black arrowheads). **(B)** wt, Klk5^-/-^Sp5^A135X/A135X^, and Klk5^-/-^Klk7^-/-^Sp5^A135X/A135X^ mice at P5. Klk5^-/-^Sp5^A135X/A135X^ pups showed dry, scaly skin while Klk5^-/-^Klk7^-/-^Sp5^A135X/A135X^ mice had stretched and shiny epidermis, with no visual signs of dehydration. **(C)** wt and Klk5^-/-^Klk7^-/-^Sp5^A135X/A135X^ mice at 3 weeks. Klk5^-/-^Klk7^-/-^Sp5^A135X/A135X^ mice showed alopecia and growth retardation. **(D)** wt and Klk5^-/-^Klk7^-/-^Sp5^A135X/A135X^ mice at 6 weeks. **(E)** Vibrissae hair obtained from P5 pups analysed by scanning electron microscopy. Klk5^-/-^Klk7^-/-^Sp5^A135X/A135X^ showed hair shaft abnormalities similar to bamboo hair; Scale bar, 30 μm. **(F)** Progression of body weight of wt, Klk5^-/-^Sp5^A135X/A135X^, Klk5^-/-^Klk7^-/-^Sp5^A135X/A135X^ and Klk5^-/-^Klk7^-/-^ mice, n>5, error bars represent standard errors of mean.

Similarly to Sp5^A135X/A135X^ mutants, Klk5^-/-^Sp5^A135X/A135X^, Klk7^-/-^Sp5^A135X/A135X^, and Klk5^-/-^Klk7^-/-^Sp5^A135X/A135X^ mice showed no embryonic lethality and the pups exhibited normal Mendelian ratio. Both Sp5^A135X/A135X^ and Klk7^-/-^Sp5^A135X/A135X^ pups had a fragile epidermis with numerous epidermal lesions located mainly on the abdomen and head (Fig 3A). Cutaneous defects were strongly improved in Klk5^-/-^Sp5^A135X/A135X^ mice, which exhibited only minor skin lesions, while Klk5^-/-^Klk7^-/-^Sp5^A135X/A135X^ mice showed no skin phenotype and the pups were visually indistinguishable from wt (Fig 3A). Sp5^A135X/A135X^ P0 pups also showed underdeveloped or completely absent vibrissae hairs. These hair defects were partially rescued in Klk5^-/-^Sp5^A135X/A135X^ and to a greater extent in Klk7^-/-^Sp5^A135X/A135X^ newborn pups, which exhibited slightly shorter and irregularly distributed vibrissae hairs. Klk5^-/-^Klk7^-/-^Sp5^A135X/A135X^ did not show any major abnormalities of whiskers (S4 Fig).

Inactivation of KLK7 did not affect the survival of Sp5^A135X/A135X^ mice as Klk7^-/-^Sp5^A135X/A135X^ pups died within 12 hours after birth. Interestingly, Klk5^-/-^Sp5^A135X/A135X^ mice survived until postnatal day 5 (P5) when they exhibited reduced body-size and dry skin with severe scaling throughout the body surface (Fig 3B). In contrast, simultaneous inactivation of both KLK5 and KLK7 fully rescued the lethality and Klk5^-/-^Klk7^-/-^Sp5^A135X/A135X^ survive to adulthood. At P5, the skin of Klk5^-/-^Klk7^-/-^Sp5^A135X/A135X^ appears to be more stretched and shiny in comparison to wt mice, however they show no signs of scaling (Fig 3B). Nevertheless, Klk5^-/-^Klk7^-/-^Sp5^A135X/A135X^ showed alopecia and growth retardation 2–4 weeks after birth (Fig 3C), which disappeared with age. Interestingly, scanning electron microscopy (SEM) analysis of vibrissae and pelage hairs revealed that Klk5^-/-^Klk7^-/-^Sp5^A135X/A135X^ pups from P4 –P28 develop a specific hair shaft defect that strongly resembles bamboo hair in NS patients (Fig 3E). The hair defects and growth retardation in Klk5^-/-^Klk7^-/-^Sp5^A135X/A135X^ improve with age (Fig 3D and 3F) and no major cutaneous phenotype was seen in adulthood, with the exception of minor scaling on the ears, shorter tail, and a lower body weight (Fig 3D and 3F).

### Hyperactivity of KLK5 and KLK7 is responsible for the barrier disruption of LEKTI-deficient skin

Analysis of newborn P0 mice revealed that Sp5^A135X/A135X^ and Klk7^-/-^Sp5^A135X/A135X^ have significantly decreased weight in comparison to other mutant and wt lines (S5A Fig) while no differences where observed in E18.5 dpc embryos (S5B Fig). This suggests that the weight loss in Sp5^A135X/A135X^ and Klk7^-/-^Sp5^A135X/A135X^ lines is caused by severe epidermal barrier disruption followed by rapid dehydration.

**Fig 4:**
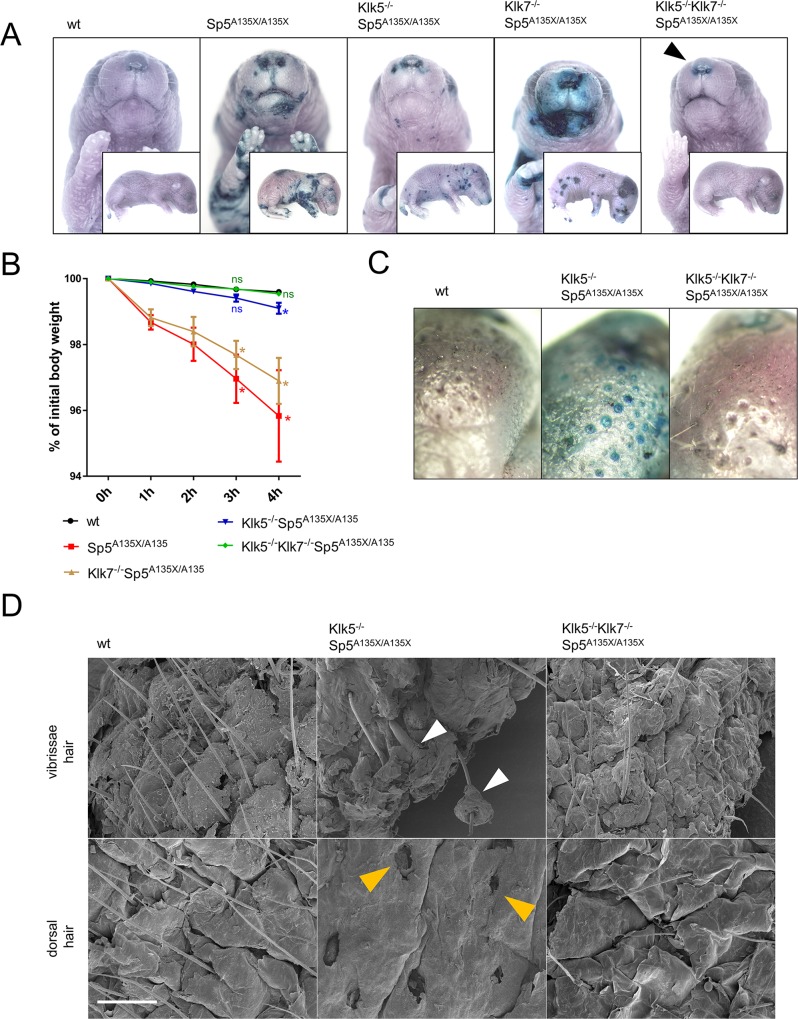
Analysis of skin-barrier abnormalities. **(A)** Newborn (P0) pups were analysed by barrier penetration assay using toluidine blue (TB). Remaining barrier-defect in the area of nostrils of Klk5^-/-^Klk7^-/-^Sp5^A135X/A135X^ pups in the vicinity of nostrils is marked by black arrowhead **(B)** TEWL analysis of P0 pups as a reduction of body-weight over time, n≥4 for each genotype. Error bars represent standard errors of mean; Klk/Spink5 mutant lines were compared with the wt line using Mann-Whitney U-test at 3h and 4h, ns means “not significant“; * p < 0.05 **(C)** Epidermal barrier in P5 Klk5^-/-^Sp5^A135X/A135X^ pups was compromised in the proximity of hair follicles **(D)** Vibrissae hair (upper panel) and dorsal skin (lower panel) obtained from P5 pups analysed using scanning electron microscopy. Defective separation of hair shafts from the root sheath in Klk5^-/-^Sp5^A135X/A135X^ is marked by white arrowhead. Dorsal skin of Klk5^-/-^Sp5^A135X/A135X^ mice showed complete absence of hair shafts and exposed upper parts of hair follicles (yellow arrowheads); Scale bar, 300 μm.

The integrity of epidermal barrier in Sp5^A135X/A135X^newborn P0 pups was analysed using the toluidine blue (TB) penetration assay and showed severe skin barrier disruption marked by penetration of TB through large areas of the body, mainly the abdomen, paws, and head. Ablation of KLK7 on the Sp5^A135X/A135X^ background did not improve the barrier and Klk7^-/-^Sp5^A135X/A135X^ newborns showed similar barrier disruption to Sp5^A135X/A135X^. In contrast, Klk5^-/-^Sp5^A135X/A135X^ newborns developed less severe barrier phenotype characterised by multiple small stained patches and in Klk5^-/-^Klk7^-/-^Sp5^A135X/A135X^ pups, the barrier integrity was almost completely recovered and the mice showed TB staining only in the area of nostrils (Fig 4A).

To confirm that disruption of skin barrier leads to the dehydration of newborn mice, we assessed the trans-epidermal water loss (TEWL) in P0 pups over time. Consistently with previous analyses of epidermal barrier properties, we found significantly impaired water retention in Sp5^A135X/A135X^ and Klk7^-/-^Sp5^A135X/A135X^. Water barrier was partially rescued in Klk5^-/-^Sp5^A135X/A135X^ pups and completely restored in Klk5^-/-^Klk7^-/-^Sp5^A135X/A135X^ (Fig 4B).

To understand why Klk5^-/-^Sp5^A135X/A135X^ died at P5, mice were stained with TB at P5. Interestingly, Klk5^-/-^Sp5^A135X/A135X^ mice showed clear penetration of the dye in the epidermis adjacent to hair shafts whereas this barrier defect was completely rescued in Klk5^-/-^Klk7^-/-^Sp5^A135X/A135X^ (Fig 4C). Detailed analysis of the epidermis using SEM revealed that P5 Klk5^-/-^Sp5^A135X/A135X^ mice had dramatic epidermal defects manifested by defective separation of hair shafts from the surrounding tissues and subsequent loss of infundibular epidermis in the upper part of hair follicles. These defects were not observed in Klk5^-/-^Klk7^-/-^Sp5^A135X/A135X^ (Fig 4D) thus suggesting that KLK7 activity is responsible for the epidermal barrier defects contributing to the lethality of Klk5^-/-^Sp5^A135X/A135X^ at P5. No barrier abnormalities were observed in P0 or P5 pups from control lines Klk5^-/-^, Klk7^-/-^ and Klk5^-/-^Klk7^-/-^.

### Both, KLK5 and KLK7 contribute to abnormal differentiation of LEKTI-deficient epidermis

**Fig 5:**
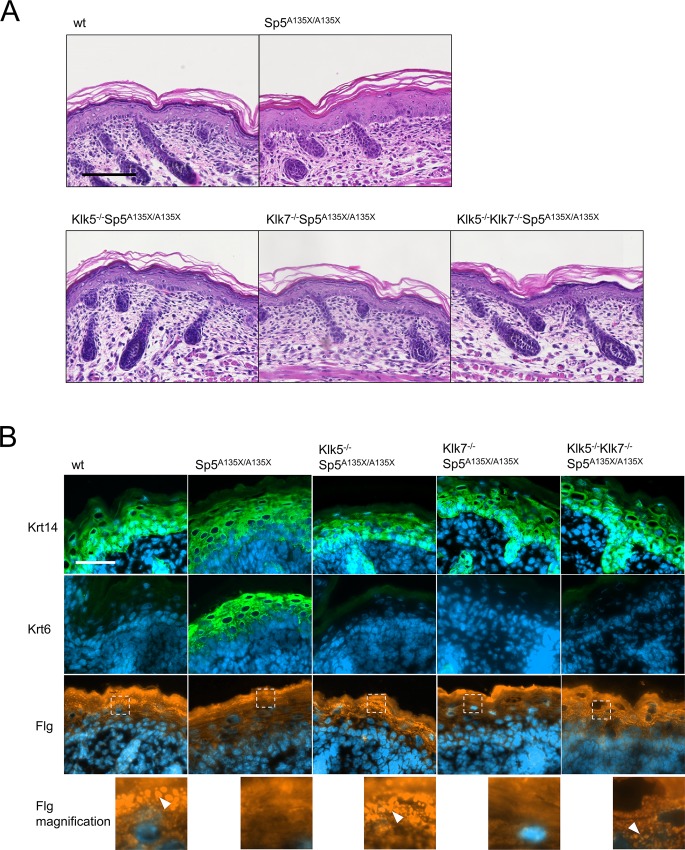
Histological analysis of epidermis structure at E18.5 dpc and P0. **(A)** Hematoxylin and eosin stained skin sections from newborn mice showed a reduced granular layer and acanthosis in epidermis of Sp5^A135X/A135X^ pups; no obvious defects were observed in the other groups, Scale bar, 100 μm. **(B)** Analysis of epidermal differentiation in the skin from E18.5 dpc embryos. Sections were stained with antibodies against keratin14 (Krt14), keratin6 (Krt6) and fillagrin (Flg). Increased expression of Krt14 was observed in Sp5^A135X/A135X^ embryos, which also strongly express the stress marker Krt6. Expression of both Krt6 and Krt14 was not altered in Klk5^-/-^Sp5^A135X/A135X^, Klk7^-/-^Sp5^A135X/A135X^ and Klk5^-/-^Klk7^-/-^Sp5^A135X/A135X^. Flg staining revealed absence of profilaggrin granules in Sp5^A135X/A135X^ and Klk7^-/-^Sp5^A135X/A135X^ embryos whereas these granules were present in wt, Klk5^-/-^Sp5^A135X/A135X^ and Klk5^-/-^Klk7^-/-^Sp5^A135X/A135X^ mice (white arrowheads). Scale bar, 50 μm.

Histological analysis of the epidermis from NS patients together with previously published data on LEKTI-deficient models describe an abnormally differentiated epidermis [15]. In line with these observations, analysis of non-lesional skin of P0 pups showed a reduced granular layer, acanthosis and sporadic SC detachment and parakeratosis in Sp5^A135X/A135X^ pups (Fig 5A and S6 Fig). Although Klk7^-/-^Sp5^A135X/A135X^ pups showed a similar phenotype to Sp5^A135X/A135X^ mice, no differentiation defects were observed in the epidermis of newborn pups apart from the occasional focal detachment of SC (Fig 5A and S6B Fig). Klk5^-/-^Sp5^A135X/A135X^ and Klk5^-/-^Klk7^-/-^Sp5^A135X/A135X^ newborn pups exhibited well differentiated epidermal layers.

To address the characteristics of epidermal differentiation defects, we analysed the expression of several differentiation markers in E18.5 dpc embryos to avoid the contribution of secondary effects following barrier disruption and exposure to the environment (Fig 5B). In accordance with previous results, Sp5^A135X/A135X^ embryos exhibited a poorly defined basal layer, abnormal expression of keratin 14 (Krt14) and markedly increased expression of keratin 6 (Krt6), suggesting hyperproliferation of keratinocytes in Sp5^A135X/A135X^ embryos (Fig 5B). Krt14 and Krt6 expression in Klk5^-/-^Sp5^A135X/A135X^, Klk7^-/-^Sp5^A135X/A135X^, and Klk5^-/-^Klk7^-/-^Sp5^A135X/A135X^ embryos exhibited a similar pattern to wt animals, indicating that the differentiation defects of LEKTI-deficient epidermis are fully dependent on concurrent activities of both KLK5 and KLK7. In the epidermis of both Sp5^A135X/A135X^ and Klk7^-/-^Sp5^A135X/A135X^ embryos profilaggrin granules were absent whereas they were present in Klk5^-/-^Sp5^A135X/A135X^, Klk5^-/-^Klk7^-/-^Sp5^A135X/A135X^ and in wt mice (Fig 5B).

**Fig 6:**
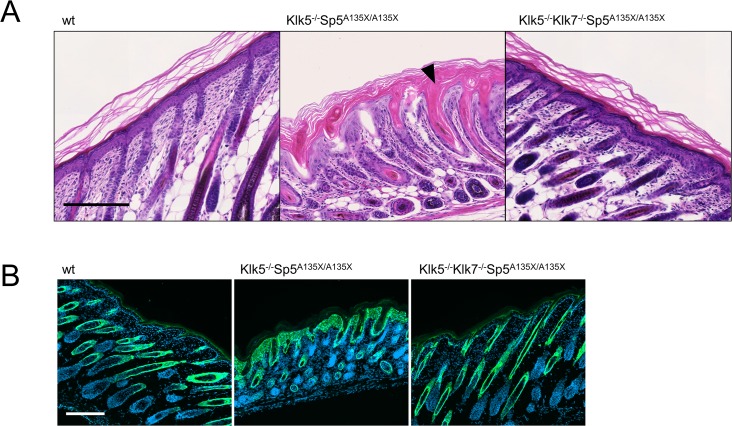
Histological analysis of epidermis structure at P5. **(A)** Hematoxylin and eosin stained dorsal skin from wt, Klk5^-/-^Sp5^A135X/A135X^, and Klk5^-/-^Klk7^-/-^Sp5^A135X/A135X^ mice at P5. Klk5^-/-^Sp5^A135X/A135X^ pups show acanthosis and both the distribution and orientation of hair follicles were distorted. The follicles exhibit severe defects manifested by severe hyperkeratosis of isthmus and infundibulum (black arrowhead). The skin in Klk5^-/-^Klk7^-/-^Sp5^A135X/A135X^ mice was comparable to wt mice. Scale bar, 200 μm **(B)** Skin sections from P5 pups were stained using anti-Keratin6 antibody. Klk5^-/-^Sp5^A135X/A135X^ show hyperproliferation of keratinocytes, which is rescued in Klk5^-/-^Klk7^-/-^Sp5^A135X/A135X^. Scale bar, 200 μm.

As Klk5^-/-^Sp5^A135X/A135X^ exhibited drastic epidermal defects before they die at P5 (Fig 4C and 4D), we performed histological analysis of skin from Klk5^-/-^Sp5^A135X/A135X^, Klk5^-/-^Klk7^-/-^Sp5^A135X/A135X^ and wt mice at P5. We found that although Klk5^-/-^Sp5^A135X/A135X^ pups showed normal differentiation at P0, over time they developed hyperplastic epidermis with acanthosis, severe intrafollicular hyperkeratosis and the skin was infiltrated by mast cells (Fig 6A and S7 Fig). In contrast, Klk5^-/-^Klk7^-/-^Sp5^A135X/A135X^ showed no such defects and the epidermis was comparable to wt (Fig 5C). We also observed an increased expression of keratin6 in the epidermis of Klk5^-/-^Sp5^A135X/A135X^ P5 pups, which was rescued in Klk5^-/-^Klk7^-/-^Sp5^A135X/A135X^ (Fig 6B). Analysis of corneodesmosomal proteins revealed markedly decreased expression of CDSN at the *stratum corneum*/*stratum granulosum* interface in Klk5^-/-^Sp5^A135X/A135X^, but not in Klk5^-/-^Klk7^-/-^Sp5^A135X/A135X^ pups (S8 Fig). These data suggest that abnormal epidermal differentiation of Klk5^-/-^Sp5^A135X/A135X^ P5 pups is caused by KLK7 activity.

### Inflammation of LEKTI-deficient skin depends on KLK5, KLK7 and is modulated by age

**Fig 7:**
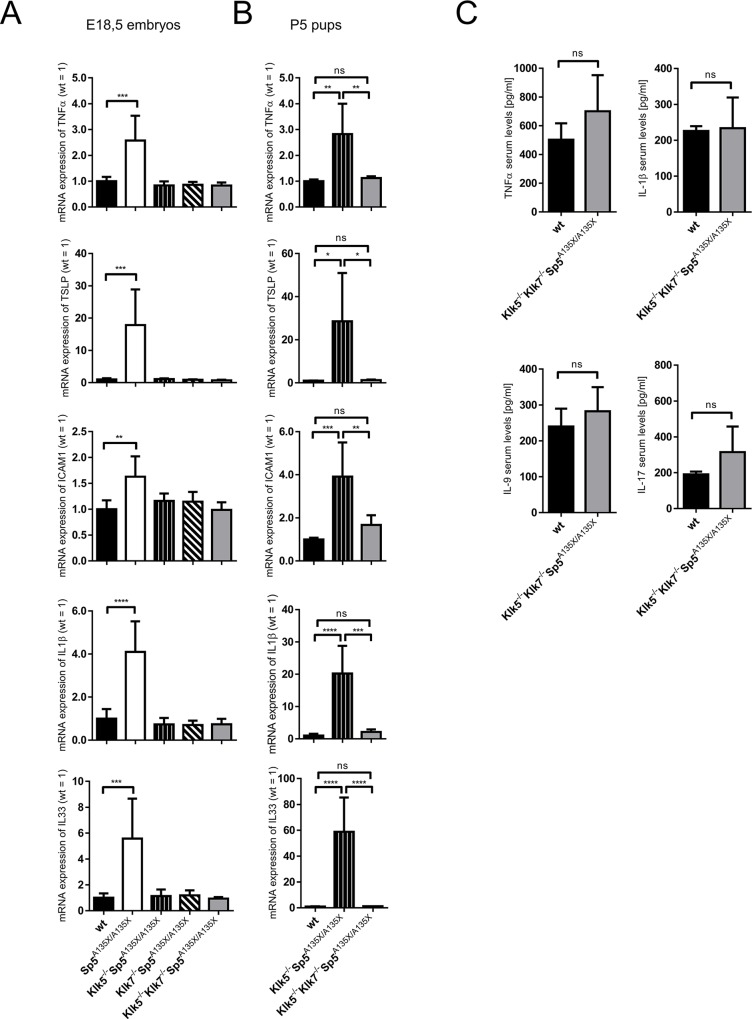
Analysis of cutaneous and systemic inflammation. (A) Expression of TSLP, TNFα, ICAM1, IL-1β and IL33 in the skin of E18.5 dpc wt, Sp5^A135X/A135X^, Klk5^-/-^Sp5^A135X/A135X^, Klk7^-/-^Sp5^A135X/A135X^, and Klk5^-/-^Klk7^-/-^Sp5^A135X/A135X^ analysed by qRT-PCR, n≥5. Error bars represent standard deviations from mean; Data were analysed by One-way ANOVA followed by Bonferroni post-hoc tests. (B) Cytokine expression in the skin of P5 wt, Klk5^-/-^Sp5^A135X/A135X^, Klk5^-/-^Klk7^-/-^Sp5^A135X/A135X^ and Klk5^-/-^ analysed by qRT-PCR, n≥3. Error bars represent standard deviations from mean; Data were analysed by One-way ANOVA followed by Bonferroni post-hoc tests. (C) Analysis of serum levels of TNFα, IL-1β, IL-9 and IL-17 in 6 weeks old wt and Klk5^-/-^Klk7^-/-^Sp5^A135X/A135X^ mice, n≥4. Error bars represent standard deviations from mean; Means were compared with a *t-*test, ns means “not significant“; * p < 0.05; ** p < 0.01; *** p < 0.001; **** p < 0.0001.

As the aggravated inflammatory response and allergic manifestations are symptomatic for NS, we assayed the expression of pro-inflammatory and pro-TH2 cytokines in the skin isolated from Spink5- and Klk-deficient E18.5 dpc embryos. As expected, Sp5^A135X/A135X^ embryos showed elevated expression levels of TNFα, TSLP, Il-33, Il-1β as well as ICAM1 (Fig 7A). In contrast, these cytokines were not upregulated in Klk5^-/-^Sp5^A135X/A135X^ animals, which is in line with the recent study of Furio *et al*. [24] indicating that KLK5 is responsible for triggering the inflammation in LEKTI-deficient epidermis. However, expression levels of TNFα, TSLP, Il-33, Il-1β and ICAM1 were also completely normal in mice with ablated KLK7, i.e. in Klk7^-/-^Sp5^A135X/A135X^ (Fig 7A). Full rescue of cutaneous inflammation was also observed in Klk5^-/-^Klk7^-/-^Sp5^A135X/A135X^ embryos (Fig 7A).

To analyse cutaneous inflammation in later stages of development, we assayed expression levels of TNFα, TSLP, Il-33, Il-1β and ICAM1 in the skin of P5 pups from surviving LEKTI-deficient mutant lines Klk5^-/-^Sp5^A135X/A135X^ and Klk5^-/-^Klk7^-/-^Sp5^A135X/A135X^ and wt controls. Although no signs of cutaneous inflammation were found in Klk5^-/-^Sp5^A135X/A135X^ E18.5 dpc embryos, P5 pups showed significant upregulation of TNFα, TSLP, Il-33, Il-1β and ICAM1 (Fig 7B). Expression of these cytokines was normalized by inactivation of KLK7, in Klk5^-/-^Klk7^-/-^Sp5^A135X/A135X^ P5 pups (Fig 7B).

NS is also associated with systemic inflammation, allergy and elevation of IgE levels. We examined the serum levels of TNFα, Il-1β, IL-9 and IL-17 in Klk5^-/-^Klk7^-/-^Sp5^A135X/A135X^ 6 weeks old mice, however no signs of systemic inflammation were found when compared to wt mice (Fig 7C). These observations are in line with the rescue of other NS-like symptoms in adult Klk5^-/-^Klk7^-/-^Sp5^A135X/A135X^ mice.

## Discussion

Netherton syndrome is a severe genetic disorder associated with unregulated proteolytic activity, caused by the absence of functional LEKTI, a protease inhibitor encoded by *SPINK5* gene. In this study, we elucidated the roles of KLK5 and KLK7 proteases in the disease by genetic inactivation of these proteases on the background of a mouse model for NS. This novel model was generated by mimicking a SPINK5 p.A134X mutation found in human patients [25] and recapitulates the phenotype of previously described *Spink5*-deficient mouse models [15,26,27]. However, our Sp5^A135X/A135X^ mice in combination with the ablation of KLK5 and KLK7 reveal the complexity of the LEKTI-KLK network. We showed, that although single inactivation of KLK5 or KLK7 rescues a number of NS-like pathological manifestations, only simultaneous ablation of both proteases fully rescues the lethal phenotype of Sp5^A135X/A135X^ mice.

It has been proposed that the barrier defects observed in LEKTI-deficient skin are caused by proteolytic hyperactivity leading to premature degradation of corneodesmosomal proteins. *In vitro* assays showed that three putative LEKTI targets are able to promote corneodesmosome degradation namely KLK5, KLK7, and KLK14 [18]. The *in vitro* study of the KLK proteolytic activation cascade proposed that KLK5 acts upstream from KLK7 and KLK14 and therefore, KLK5 hyperactivity should contribute to barrier defects either directly or indirectly via activation of the remaining KLKs. Indeed, significant improvement of skin-barrier defects by inactivation of KLK5 in Sp5^A135X/A135X^ mice was observed, however the rescue was incomplete as toluidine blue staining in Klk5^-/-^Sp5^A135X/A135X^ mice revealed patches of disrupted-barrier distributed all over the body surface. This observation implicates the role of another protease whose activity contributes to barrier defects in the absence of LEKTI and does not depend on KLK5. This was identified as KLK7, since Klk5^-/-^Klk7^-/-^Sp5^A135X/A135X^ newborn mice did not show any major barrier defects of epidermis. Interestingly, single inactivation of KLK7 on Sp5^A135X/A135X^ background did not significantly improve the barrier defects. Therefore we assume that barrier properties of LEKTI-deficient neonatal epidermis are compromised mainly by direct activity of KLK5 and only to a lesser extent by KLK5-mediated activation of KLK7. The significant contribution of KLK5 to NS pathology is in line with a recent study of Furio et al. showing amelioration of skin barrier-phenotype in Spink5-deficent newborns upon KLK5 inactivation [24]. Nevertheless, the remaining activity of KLK7 still contributes to the defective barrier and further intensifies with age as Klk5^-/-^Sp5^A135X/A135X^ show severe epidermal defects manifested by loss of infundibular epidermis at P5. Klk5^-/-^Klk7^-/-^Sp5^A135X/A135X^ mice exhibit no skin-barrier defects at P5 and most importantly, in contrast to Klk5^-/-^Sp5^A135X/A135X^ mice, the triple mutants survive to adulthood. The skin defects in Klk5^-/-^Sp5^A135X/A135X^ P5 pups markedly resemble those observed in mice deficient for corneodesmosomal proteins CDSN and DSC1 [30,31]. Indeed, Klk5^-/-^Sp5^A135X/A135X^ show reduced CDSN expression at P5, which indicates that unregulated activity of KLK7 results in degradation of corneodesmosomes. The mechanism of pro-KLK7 activation in the absence of KLK5 remains unclear. KLK7 can be activated by matriptase [32] and a recent study also suggests a role of mesotrypsin in pro-KLK7 activation [33].

Although the lethal phenotype is fully rescued in Klk5^-/-^Klk7^-/-^Sp5^A135X/A135X^ mutants and the mice do not show any signs of skin barrier-defects leading to dehydration, we observed minor barrier disruptions in the nostril area of newborn pups, which suggests the activity of another protease physiologically inhibited by LEKTI. As the toluidine blue -stained area overlaps with the expression of KLK14 in late embryonic development (S9A Fig), we propose that KLK14 could be responsible for the remaining pathology of LEKTI-deficient mice even in the absence of KLK5 and KLK7. Moreover, we and others also observed expression of KLK14 in hair follicles (S9B Fig)[34], which makes KLK14 a candidate protease responsible for the development of the bamboo hair defect in Klk5^-/-^Klk7^-/-^Sp5^A135X/A135X^ animals up to the age of 3 weeks. As reported, the defects of cell adhesion proteins in hair follicles result in “lanceolate hair”–a hair shaft phenotype in mice that strongly resembles the bamboo hairs of Klk5^-/-^Klk7^-/-^Sp5^A135X/A135X^ mutants and NS patients [35,36]. This further supports a possible role of KLK14 in the formation of bamboo-hair, as KLK14 is linked to the degradation of desmosomal proteins in LEKTI-deficient epidermis [8]. Nevertheless, any targets of LEKTI inhibition present in hair follicles, such as caspase-14 [37] or other, currently unidentified proteases, should be considered as a potential cause of bamboo hairs.

Association of NS with abnormal epidermal differentiation accompanied by acanthosis, parakeratosis, and hyperproliferation of keratinocytes was previously reported in Spink5-deficient mouse models [15,26,27] and our Sp5^A135X/A135X^ confirms the previous findings. We found clear overexpression of keratin6 in Sp5^A135X/A135X^ E18.5 dpc epidermis, suggesting that events leading to hyperproliferation of keratinocytes are triggered prior to the exposure to the external environment and are a result of unregulated proteolytic activity in the epidermis. In light of the fact that single inactivation of either KLK5 or KLK7 completely rescues the differentiation defects in LEKTI-deficient embryos as well as in newborn mice, we believe that the signalling events resulting in keratinocyte hyperproliferation in neonates depend on the presence of both, KLK5 and KLK7 together. Moreover, we observed that aggravated cutaneous inflammation, which is found in E18.5 Sp5^A135X/A135X^ embryos, fully depends on simultaneous activity of both, KLK5 and KLK7. KLK5 was previously shown to initiate inflammation in LEKTI-deficient epidermis by activation of PAR2, which results in the induction of pro-Th2 and pro-inflammatory cytokines [22–24]. In this study we show that KLK7 is also required for the induction of inflammation in LEKTI-deficient mice as P5 Klk5^-/-^Sp5^A135X/A135X^ pups developed severe acanthosis together with significantly increased expression of TNFα, TSLP, Il-33, Il-1β and ICAM1 while Klk5^-/-^Klk7^-/-^Sp5^A135X/A135X^ showed no major defects in the epidermis and increased levels of pro-inflammatory cytokines. Altogether, this suggests that inflammation and differentiation changes in older LEKTI-deficient pups (P5) are initiated by KLK7 activity which is independent of KLK5. Indeed, KLK7 was previously shown to induce inflammation and keratinocyte proliferation in the epidermis [38,39] and a recent study identified KLK7 as a proliferative factor in a mouse model of colon cancer and in human cells *in vitro* [40]. The mechanism by which KLK7 induces inflammation and differentiation changes remains to be elucidated. In contrast to KLK5, KLK7 cannot directly activate PAR2 as shown *in vitro* [41] and thus, the inflammation is likely to be triggered by a different mechanism. One possible pathway is the KLK7-mediated conversion of pro-IL1β to active IL1β [42], which could affect the inflammatory phenotype of NS- epidermis.

In summary, we show that the individual inactivation of KLK5 or KLK7 only partially rescues the defective skin barrier but not the lethal phenotype of Sp5^A135X/A135X^. Only the concurrent ablation of both KLK5 and KLK7 can fully rescue the lethal phenotype of Sp5^A135X/A135X^ mice, therefore both proteases should be investigated as clinical targets. We show that KLK7 plays an important role in the inflammation and defective differentiation in NS and its activity is not dependent on activation by KLK5. We also show that the pathological effects of unregulated KLK activities are remarkably age dependent. Altogether, this study expounds the complexity of the proteolytic network and its regulation, which are especially important to understand Netherton syndrome and its treatment.

## Materials and Methods

### Generation of targeted mice

All animal studies were ethically reviewed and performed in accordance with European directive 2010/63/EU and were approved by the Czech Central Commission for Animal Welfare.

### Generation of Klk5^-/-^ mice

Knock-out first allele of *Klk5* was produced by introduction of targeting construct (vector PRPGS00082_A_A10 obtained from NIH Knock-out Mouse Program, KOMP) via homologous recombination in embryonic stem cells (ESC). Positively targeted ESC were injected into developing wt embryos, to produce chimeric mice, which were used to establish Klk5^-/-^ line.

### Generation of Sp5^A135X/A135X^ mice

TALENs targeting exon5 of *Spink5* gene were designed using TAL Effector Nucleotide Targeter 2.0 (https://tale-nt.cac.cornell.edu/) [43,44], assembled using the Golden Gate Cloning system[43], and cloned into the ELD-KKR backbone plasmid as described previously [45]. DNA binding domains of TALENs specific for the desired target site within *Spink5* gene (Fig 1B) consisted of following repeats: NI-HD-HD-NN-HD-NI-NN-NG-NI-NN-NI-NG-NN-NG-NN-NI-NI-HD-NG (5´ TALEN-Spink5) and HD-NG-NN-HD-NG-NG-NG-NI-NG-NI-NN-NN-NN-NG-NI-HD-NG-HD-NI-HD (3´ TALEN-Spink5). Both TALEN plasmids were used for production of TALEN encoding mRNA as described previously [46]. 5 μl of TALEN mRNA (with total RNA concentration of 40 ng/μl) was mixed with 100 μM of targeting single-stranded oligonucleotide (Sigma-Aldrich; Fig 1B) and the final solution was microinjected into C57BL6/N-derived zygotes. Genomic DNA isolated from tail biopsies of newborn mice were screened by PCR (primers F1: 5´-CCTGTCTCTGCCTTCAGACC-3´ and R1: 5´-GGCTGTGGTAACTGTCCAAAA-3´) and subsequent RFLP analysis using *Xba*I restriction enzyme (Thermo-Scientific).

### Generation of Klk5^-/-^Klk7 ^-/-^ mice

TALENs were designed and synthesized as described above. DNA binding domains of TALENs specific for exon3 of murine *Klk7* contained following repeats: NN-NG-NI-NI-NI-NN-NI-NI-NN-NN-HD-NG-HD-NN-HD (5´ TALEN-Klk7) and NN-NI-NG-NG-NN-HD-HD-NG-NG-NG-NN-NI-NN-HD-NI-NN (3´ TALEN-Klk7). TALEN mRNA with total RNA concentration of 40 ng/μl was microinjected into Klk5^-/-^oocytes. Genomic DNA isolated from tail biopsies of newborn mice was screened by PCR (primers F3: 5´- GGAGAAGGCCAGGGTCTGAA-3´ and R3: 5´- TGGTCAGAAACCCACGGAGA-3´) and subsequently analyzed by RFLP using *Nco*I restriction enzyme (Thermo-Scientific).

### TEWL measurement

Newborn pups from at least two independent litters were separated from mothers to prevent fluid intake. The rate of water loss was analyzed by measuring the reduction of initial body weight at 1h, 2h, 3h and 4 h.

### Barrier penetration assay

Newborn mice were euthanized and then dehydrated by incubation for 5 min in 25, 50, 75, and 100% methanol. After rehydration in PBS, mice were incubated for 4 hours in 0.1% toluidine blue (Sigma-Aldrich), washed in PBS and imaged.

### Histology

Newborn pups or skin tissues were fixed in 3.6% formaldehyde for 24 h and embedded in paraffin. 5-μm sections were prepared using microtome were stained by hematoxylin/eosin (H&E) or by 0.5% toluidine blue using standard protocols. Images were obtained using Zeiss Axioscan Z1 (Carl Zeiss AG).

### Immunohistochemistry

Dorsal skin of P5 pups was fixed in 3.6% formaldehyde for 24 hours and embedded in paraffin. 5-μm paraffin sections were used for antigen retrieval with Discovery Ultra automated IHC/ISH system (Ventana) and stained with antibodies against Dsg-1 (Santa Cruz, 1:100 dilution, retrieval at pH6) and CDSN (Abcam, 1:100 dilution, retrieval at pH6). After 1 hour incubation at room temperature, anti-rabbit peroxidase conjugated polymer (Zytomed GmBH) was applied for 30 min and the reaction was developed using DAB (DAKO) as a chromogen. Images were obtained using Zeiss Axioscan Z1 (Carl Zeiss AG).

In order to stain the cryosections, dorsal skin of E18.5 dpc embryos or P5 pups was isolated, embedded in Tissue-Tek O.C.T (Sakura), and frozen at -80°C. 6 μm-sections were stained as described previously [47], using antibodies against Keratin6 (Covance, 1:1000 dilution), Keratin14 (Covance, 1:2000 dilution) and Filaggrin (Covance, 1:1000 dilution). Nuclei were stained using DAPI (Roche). Images were obtained using Zeiss Axioimager Z2 (Carl Zeiss AG).

### Gene expression analyses

Dorsal skin was obtained from newborn pups, crushed in liquid nitrogen and total RNA was isolated using TRIzol (Thermo-Scientific) according to the manufacturer's instructions. Residuals of genomic DNA were removed using 1 U of DNAse I (Roche) per 1 μg of RNA by 15 min incubation at 37°C. 1 μg of total RNA was used for reverse transcription by M-MLV Reverse Transcriptase (Promega) using oligo (dT) primers. RT-PCR was performed in a 20-μl reaction mixture containing SYBR Green JumpStart™ Taq ReadyMix with MgCl_2_ (Sigma-Aldrich) and 0.25mM of each primer. Respective gene expression was normalized to the expression of TATA-binding protein (TBP). Normalized expression levels were then re-expressed relative to the mean expression level of the respective target in the wt mice. Primer sequences are detailed in S1 Table.

### Scanning electron microscopy

The samples on cellulose filter paper strips were fixed with 3% glutaraldehyde in cacodylate buffer overnight at 4°C. After fixation, extensively washed samples were dehydrated through ascending alcohol concentrations followed by absolute acetone and critical point drying from liquid CO_2_ in a K 850 unit (Quorum Technologies Ltd). The dried samples were sputter-coated with 20 nm of gold in a Polaron Sputter-Coater (E5100) (Quorum Technologies Ltd). The final samples were examined in a FEI Nova NanoSem 450 scanning electron microscope (FEI) at 5 kV using secondary electron detector.

### Multiplex ELISA

The levels of TNFα, IL-1β, IL-9, and IL-17 in mouse serum were analyzed using Bio-Plex Pro Mouse Cytokine Assay (Bio-Rad Laboratories) with high sensitivity range standard settings according to manufacturer’s instructions.

## Supporting Information

S1 Fig Sequence of mutation introduced in Sp5^A135X/A135X^ mice. Successful targeting was confirmed by sequencing of cDNA obtained from Sp5^A135X/A135X^ pups, mutation present in Sp5^A135X/A135X^ is underlined.
(TIF)

S2 Fig Organization of *Klk5* mutant alleles. **(A)** Schematic of *Klk5* knock-out allele, which is present in Klk5^-/-^, Klk5^-/-^Klk7^-/-^, Klk5^-/-^Sp5^A135X/A135X^ and Klk5^-/-^Klk7^-/-^Sp5^A135X/A135X^ mutant mice **(B)** *Klk5* (loxP) allele was generated by breeding of Klk5^-/-^Klk7^-/-^ mice with FLPe expressing strain. FLPe activity leads to excision of FRT flanked cassette present in the *Klk5* knock-out allele, which leads to the restoration of *Klk5* expression. *Klk5* (loxP) allele is present in Klk7^-/-^ and Klk7^-/-^Sp5^A135X/A135X^ mutant mice.
(TIF)

S3 Fig Targeting of *Klk7* gene. **(A)** Exon organization of *Klk7* gene. TALEN-binding sequences are marked with red underline and *Nco*I restriction site that was used to analyse the targeting is marked with black underline. **(B)** Positively targeted founders (Klk7 ex3-A, Klk7 ex3-B, Klk7 ex3-C, Klk7 ex3-D) were analyzed by sequencing of exon 3 of *Klk7*, founder Klk7 ex3-A with targeted allele containing 20 bp deletion in exon 3 was selected for further breeding to establish Klk5^-/-^Klk7^-/-^ line. **(C)** Klk7-targeting site was analyzed by sequencing; cDNA was obtained from Klk5^-/-^Klk7^-/-^ animals. **(D)** KLK7 deficiency in Klk5^-/-^Klk7^-/-^ was confirmed by western blot using anti-KLK7 antibody [48].
(TIF)

S4 Fig Detail of vibrissae hair in newborn pups. Magnification of wt, Sp5^A135X/A135X^, Klk7^-/-^Sp5^A135X/A135X^, Klk5^-/-^Sp5^A135X/A135X^, and Klk5^-/-^Klk7^-/-^Sp5^A135X/A135X^ muzzle area 12 hours after birth. Whiskers of Spink5^-/-^ pups were sparse or completely missing in comparison to wt pups. Spink5^-/-^Klk5^-/-^ pups did not show any absent hair, however hair shafts were thinner and twisted when compared to wt. Despite severe epidermal defects, vibrissae hair of Klk7^-/-^Sp5^A135X/A135X^ showed almost normal appearance apart from a reduced length and uneven distribution. No hair defects were observed in Klk5^-/-^Klk7^-/-^Sp5^A135X/A135X^ P0 animals.
(TIF)

S5 Fig Body weight of E18.5 dpc embryos and newborn pups. Body weight of 12 hours old newborn pups **(A)** and E18.5 dpc embryos **(B)** wt, Sp5^A135X/A135X^, Klk7^-/-^Sp5^A135X/A135X^, Klk5^-/-^Sp5^A135X/A135X^, and Klk5^-/-^Klk7^-/-^Sp5^A135X/A135X^ lines. Although there were no significant differences in the weight of 18.5 dpc embryos between the individual lines, newborn (P0) Sp5^A135X/A135X^ and Klk7^-/-^Sp5^A135X/A135X^ pups showed significant reduction of body weight when compared to wt mice, as well as to Spink5^+/+^ littermates. The weight of Klk5^-/-^Sp5^A135X/A135X^ and Klk5^-/-^Klk7^-/-^Sp5^A135X/A135X^ newborn mice was comparable to wt animals and to Spink5^+/+^ littermates. Error bars represent standard deviations of mean, data were analysed by One-way ANOVA followed by Bonferroni post-hoc tests. n>5, ** p < 0.01.
(TIF)

S6 Fig Additional defects of epidermal structure in P0 pups. Apart from the differentiation defects described in Fig 5A, Sp5^A135X/A135X^ P0 pups showed occasional focal parakeratosis (white arrows) **(A)** and detachment of SC (double arrow) (**B**). SC detachment was found also in Klk7^-/-^Sp5^A135X/A135X^ P0 pups. Sections were stained by hematoxylin and eosin. Scale bar, 100 μm.
(TIF)

S7 Fig Infiltration of mast cells in the skin of P5 pups. Skin sections obtained from P5 pups were stained with toluidine blue. Increased infiltration of mast cells (black arrowheads) was found in Klk5^-/-^Sp5^A135X/A135X^ mice. Scale bar, 100 μm.
(TIF)

S8 Fig Expression of DSG1 and CDSN in P5 epidermis. Skin sections obtained from P5 pups were stained with antibodies against DSG1 and CDSN. CDSN-positive staining was found in the inner root sheath (black arrowheads) and upper granular layer (asterisks) of wt and Klk5^-/-^Klk7^-/-^Sp5^A135X/A135X^. In contrast, only the inner root sheaths of non-hyperplastic hair follicles of Klk5^-/-^Sp5^A135X/A135X^ P5 pups were CDSN-positive. No apparent staining was found at *stratum granulosum/startum corneum* interface or in the hyperkeratotic follicles. Scale bar, 100 μm.
(TIF)

S9 Fig Analysis of Klk14 expression pattern. Klk14 expression was monitored using a mutant strain carrying LacZ reporter under the control of Klk14 promoter. **(A)** Analysis of Klk14 expression in 18.5 dpc embryos. Klk14 was found to be expressed in the area of nostrils (black arrowhead) **(B)** Analysis of P5 skin sections revealed specific expression of Klk14 in hair follicles.
(TIF)

S1 Table List of primers. Sequences of primers used for qRT-PCR analysis.
(DOCX)
