# Supplementary figures and images for: KLK5 and KLK7 Ablation Fully Rescues Lethality of Netherton Syndrome-Like Phenotype
Source: PLoS Genet. 2017 Jan 17;13(1):e1006566. doi: 10.1371/journal.pgen.1006566 (PMC5283769; doi:10.1371/journal.pgen.1006566)

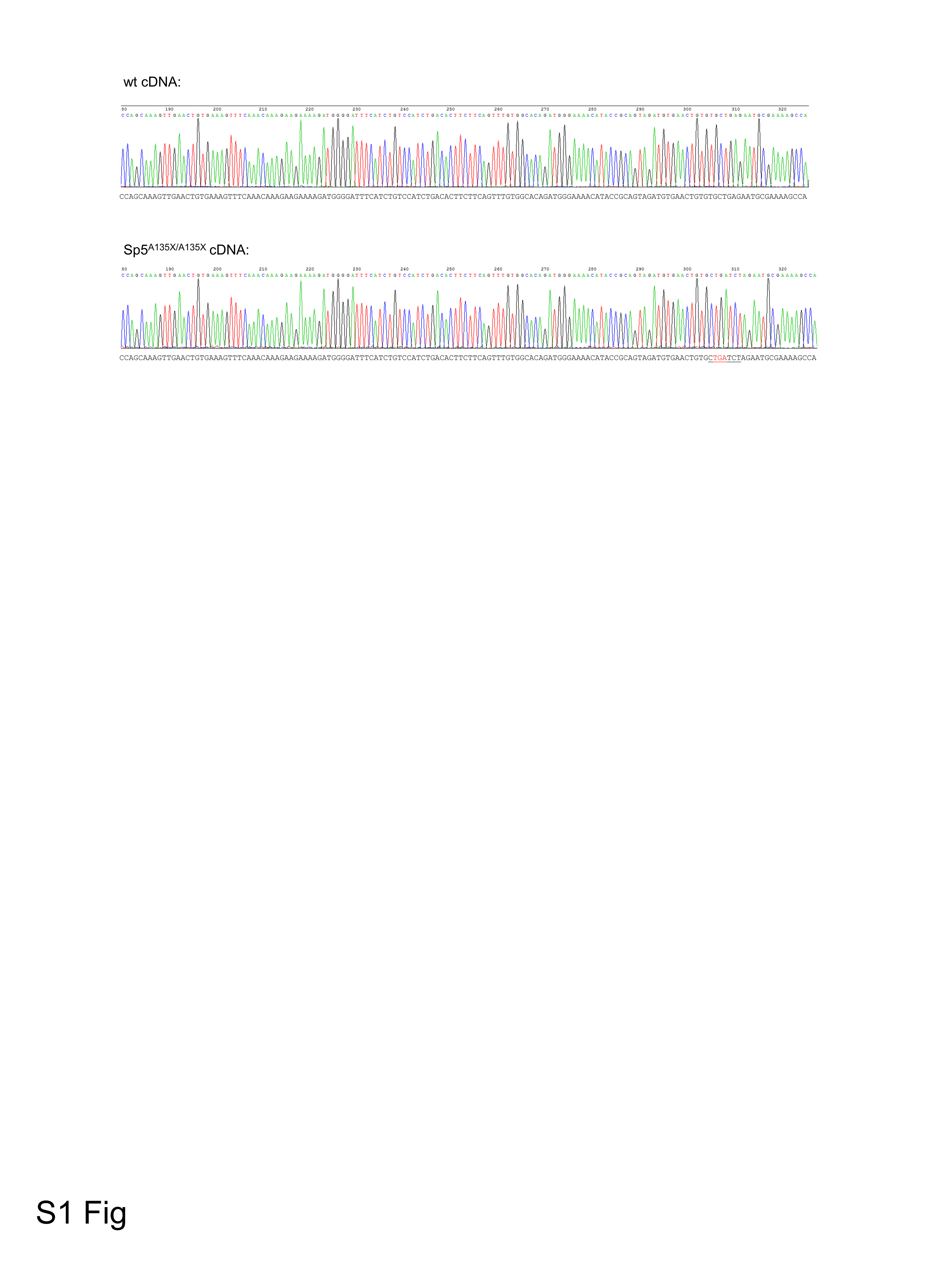

Supplement: S1 Fig — Successful targeting was confirmed by sequencing of cDNA obtained from Sp5A135X/A135X pups, mutation present in Sp5A135X/A135X is underlined. (TIF) [file pgen.1006566.s001.tif]

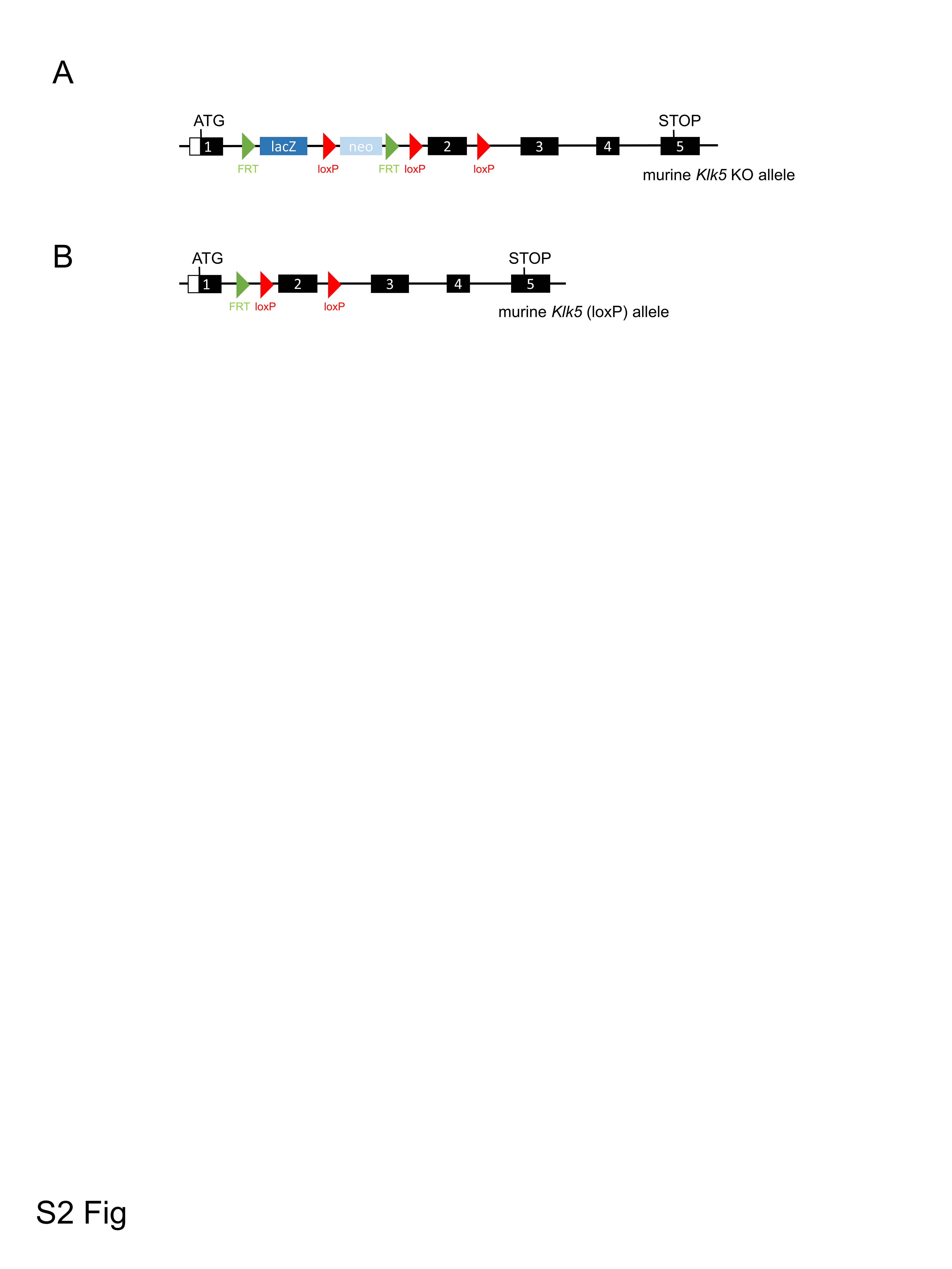

Supplement: S2 Fig — (A) Schematic of Klk5 knock-out allele, which is present in Klk5-/-, Klk5-/-Klk7-/-, Klk5-/-Sp5A135X/A135X and Klk5-/-Klk7-/-Sp5A135X/A135X mutant mice (B) Klk5 (loxP) allele was generated by breeding of Klk5-/-Klk7-/- mice with FLPe expressing strain. FLPe activity leads to excision of FRT flanked cassette present in the Klk5 knock-out allele, which leads to the restoration of Klk5 expression. Klk5 (loxP) allele is present in Klk7-/- and Klk7-/-Sp5A135X/A135X mutant mice. (TIF) [file pgen.1006566.s002.tif]

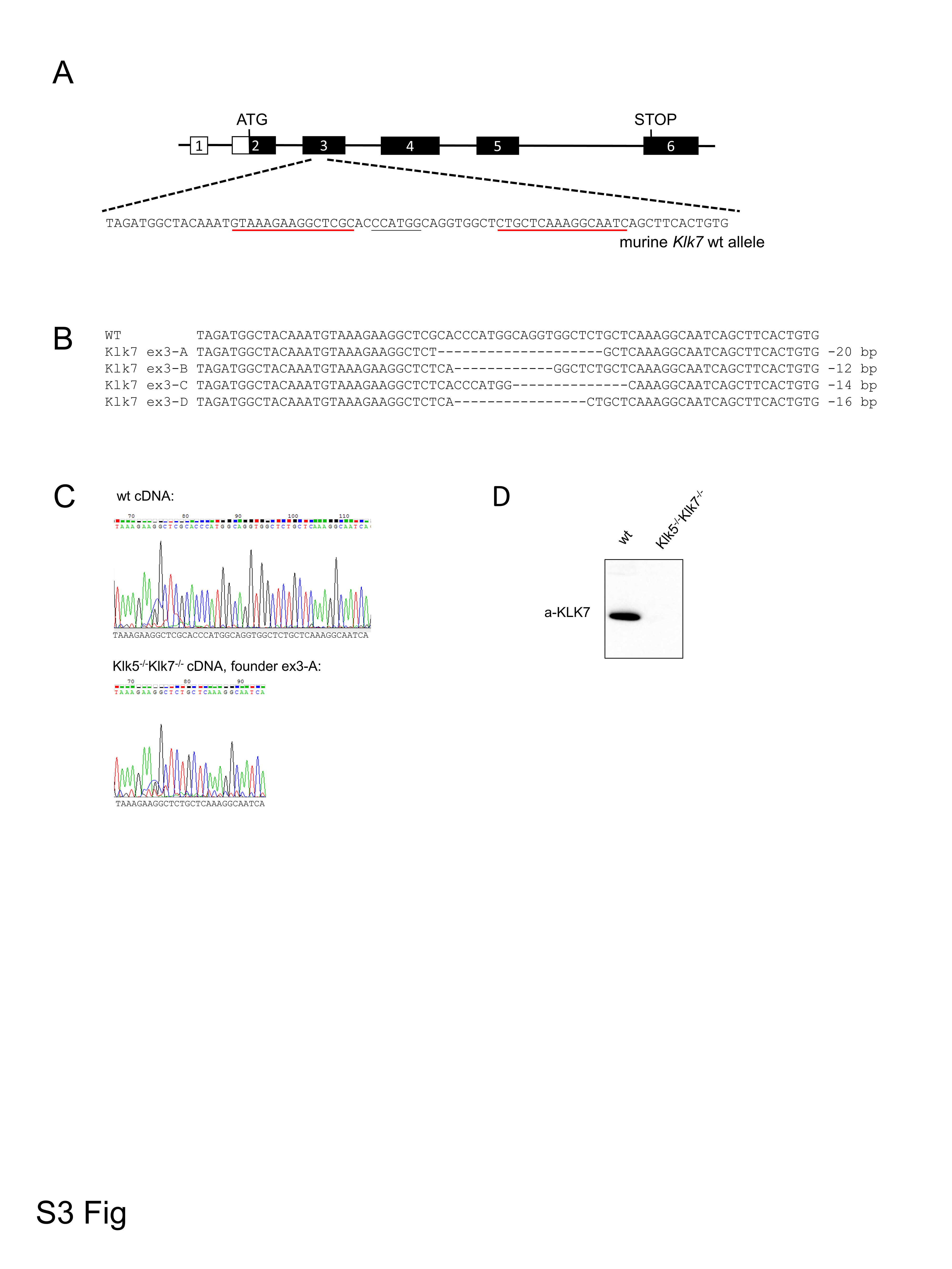

Supplement: S3 Fig — (A) Exon organization of Klk7 gene. TALEN-binding sequences are marked with red underline and NcoI restriction site that was used to analyse the targeting is marked with black underline. (B) Positively targeted founders (Klk7 ex3-A, Klk7 ex3-B, Klk7 ex3-C, Klk7 ex3-D) were analyzed by sequencing of exon 3 of Klk7, founder Klk7 ex3-A with targeted allele containing 20 bp deletion in exon 3 was selected for further breeding to establish Klk5-/-Klk7-/- line. (C) Klk7-targeting site was analyzed by sequencing; cDNA was obtained from Klk5-/-Klk7-/- animals. (D) KLK7 deficiency in Klk5-/-Klk7-/- was confirmed by western blot using anti-KLK7 antibody [48]. (TIF) [file pgen.1006566.s003.tif]

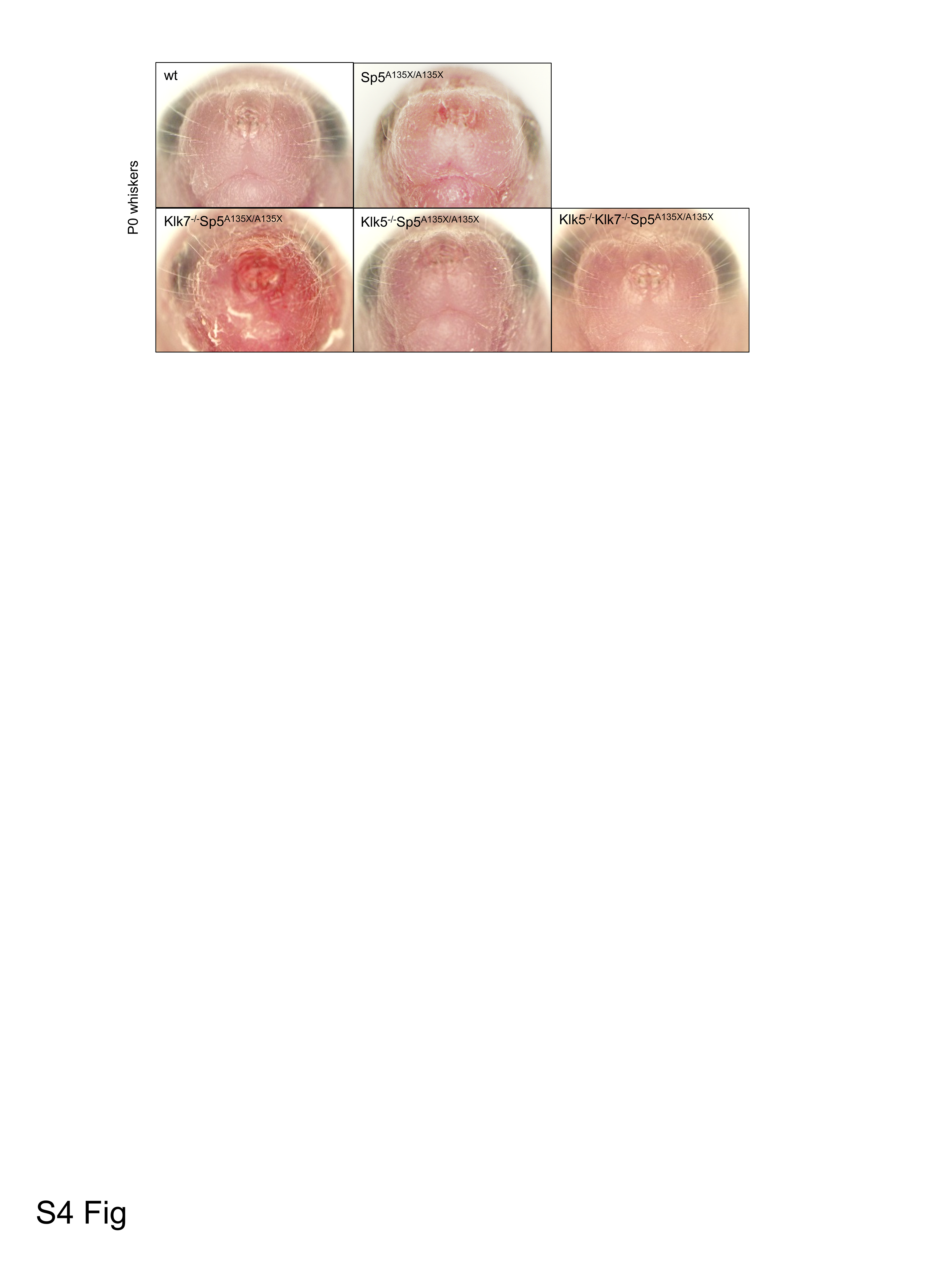

Supplement: S4 Fig — Magnification of wt, Sp5A135X/A135X, Klk7-/-Sp5A135X/A135X, Klk5-/-Sp5A135X/A135X, and Klk5-/-Klk7-/-Sp5A135X/A135X muzzle area 12 hours after birth. Whiskers of Spink5-/- pups were sparse or completely missing in comparison to wt pups. Spink5-/-Klk5-/- pups did not show any absent hair, however hair shafts were thinner and twisted when compared to wt. Despite severe epidermal defects, vibrissae hair of Klk7-/-Sp5A135X/A135X showed almost normal appearance apart from a reduced length and uneven distribution. No hair defects were observed in Klk5-/-Klk7-/-Sp5A135X/A135X P0 animals. (TIF) [file pgen.1006566.s004.tif]

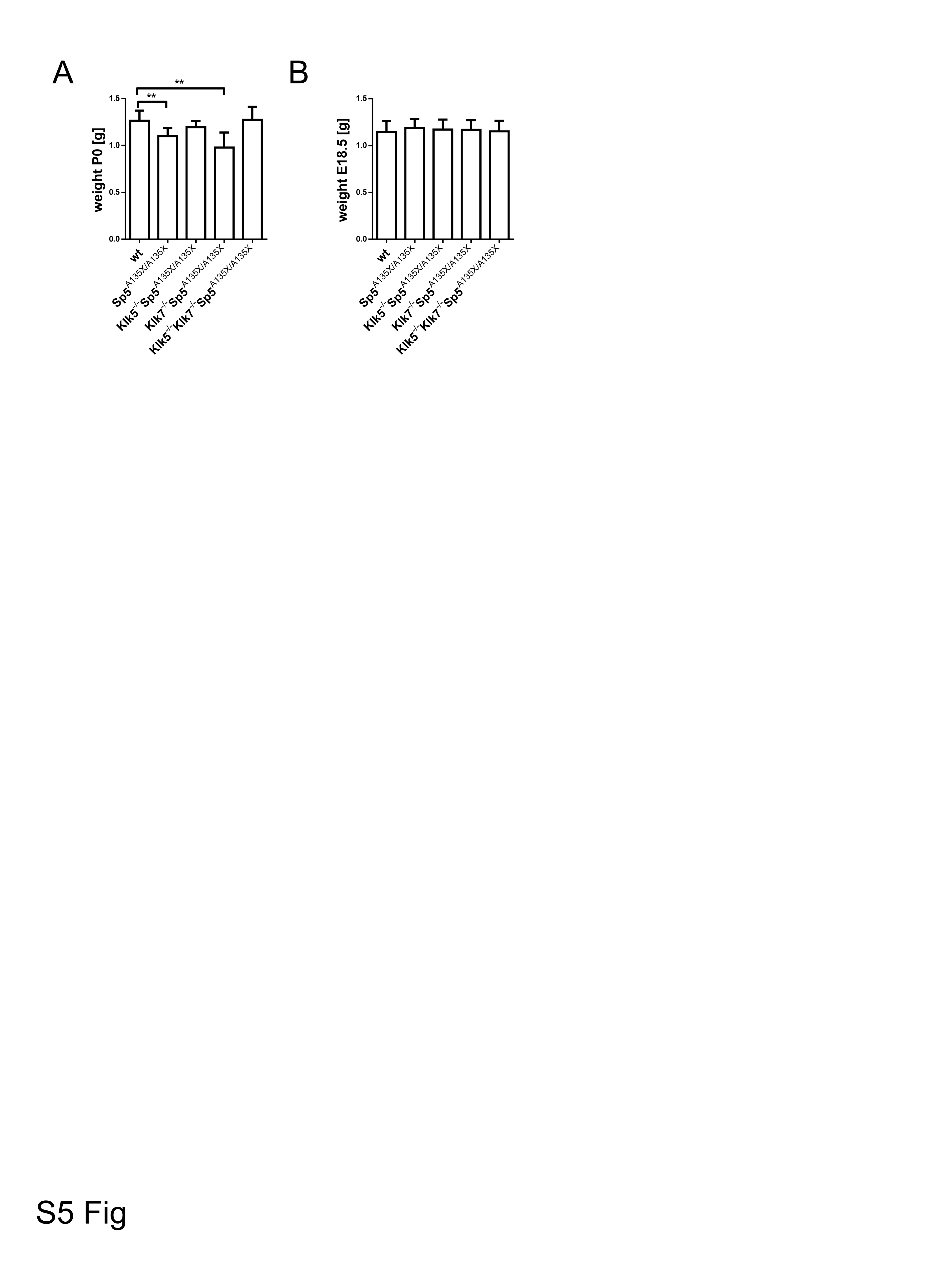

Supplement: S5 Fig — Body weight of 12 hours old newborn pups (A) and E18.5 dpc embryos (B) wt, Sp5A135X/A135X, Klk7-/-Sp5A135X/A135X, Klk5-/-Sp5A135X/A135X, and Klk5-/-Klk7-/-Sp5A135X/A135X lines. Although there were no significant differences in the weight of 18.5 dpc embryos between the individual lines, newborn (P0) Sp5A135X/A135X and Klk7-/-Sp5A135X/A135X pups showed significant reduction of body weight when compared to wt mice, as well as to Spink5+/+ littermates. The weight of Klk5-/-Sp5A135X/A135X and Klk5-/-Klk7-/-Sp5A135X/A135X newborn mice was comparable to wt animals and to Spink5+/+ littermates. Error bars represent standard deviations of mean, data were analysed by One-way ANOVA followed by Bonferroni post-hoc tests. n>5, ** p < 0.01. (TIF) [file pgen.1006566.s005.tif]

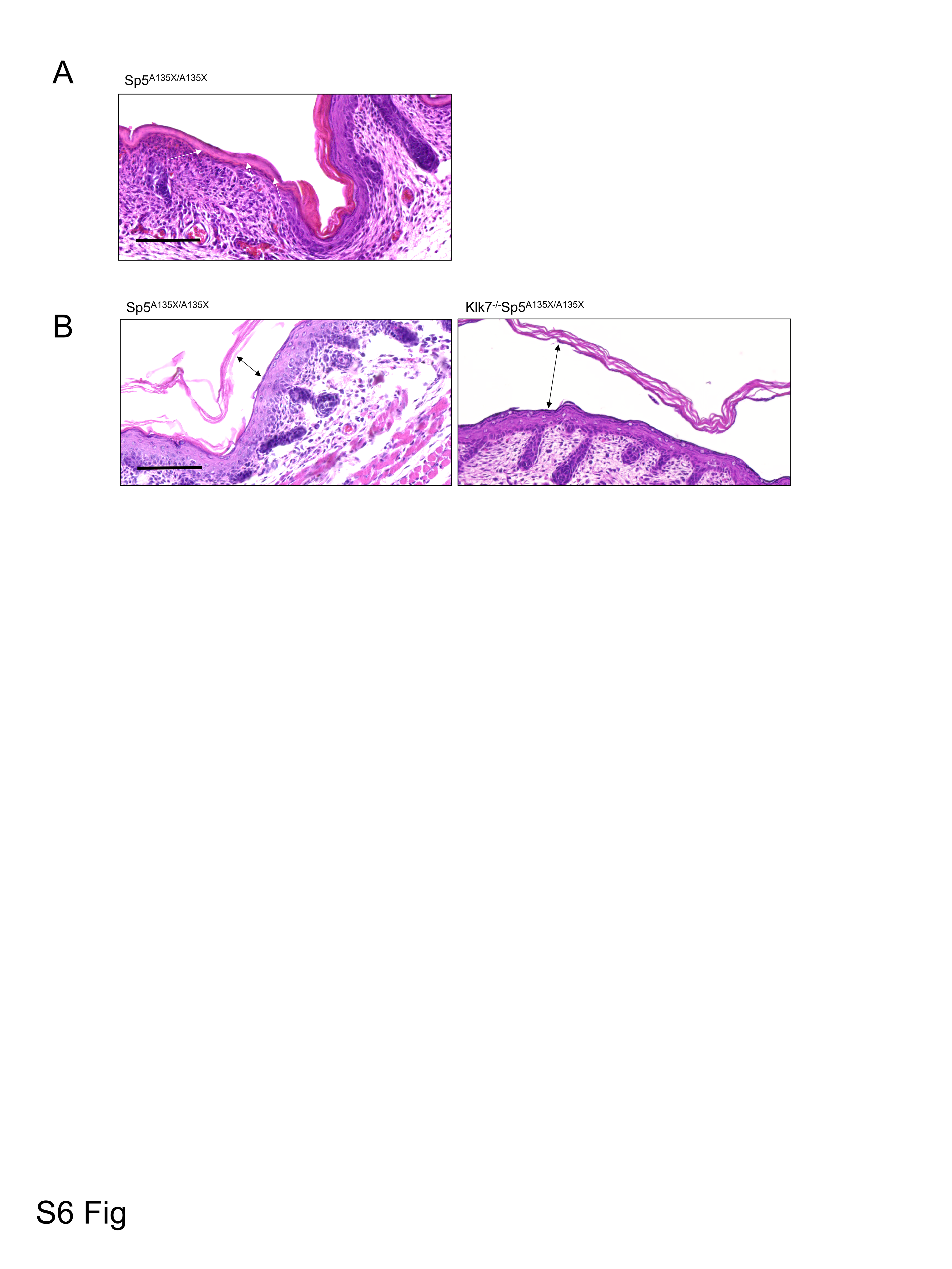

Supplement: S6 Fig — Apart from the differentiation defects described in Fig 5A, Sp5A135X/A135X P0 pups showed occasional focal parakeratosis (white arrows) (A) and detachment of SC (double arrow) (B). SC detachment was found also in Klk7-/-Sp5A135X/A135X P0 pups. Sections were stained by hematoxylin and eosin. Scale bar, 100 μm. (TIF) [file pgen.1006566.s006.tif]

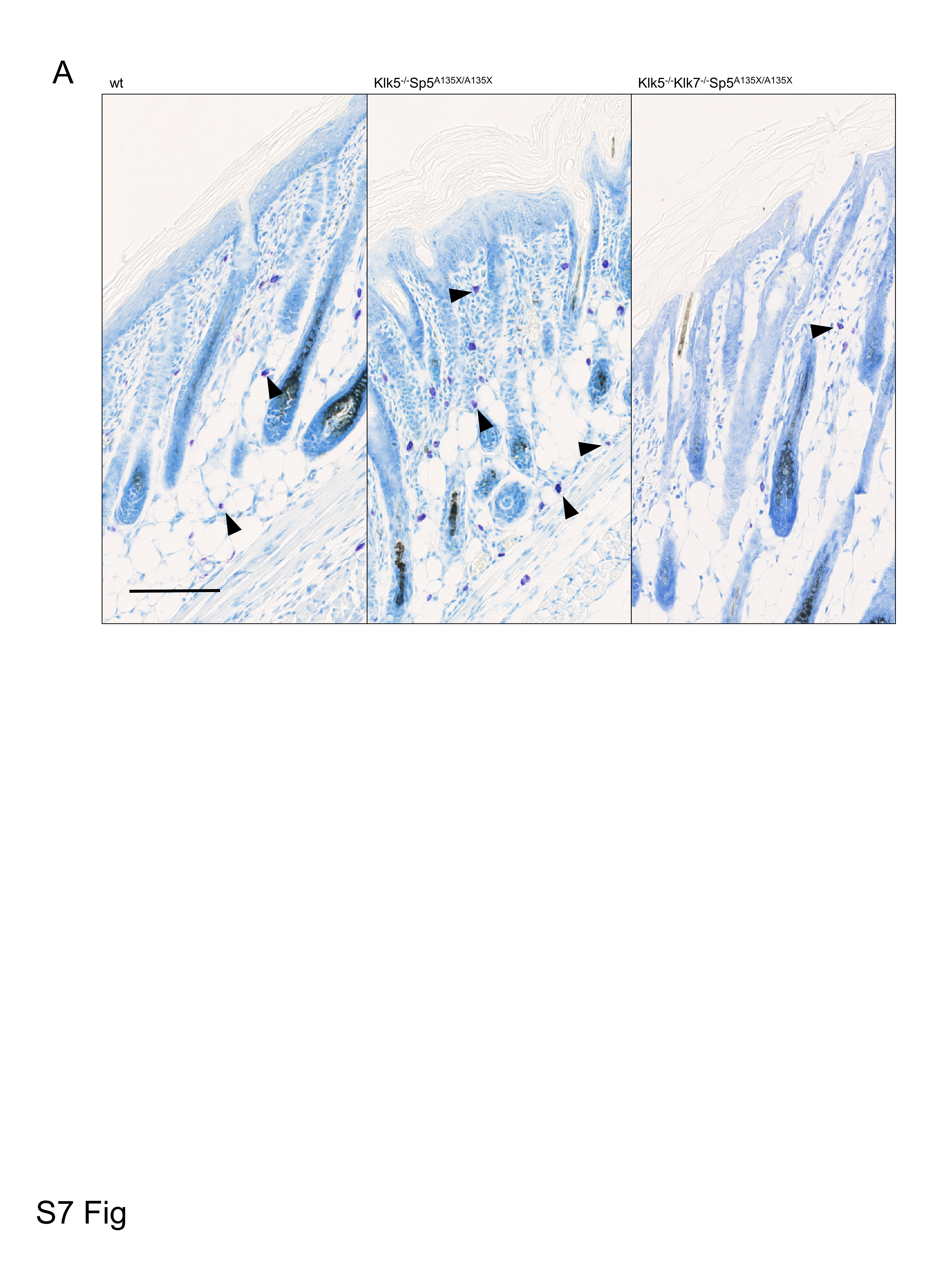

Supplement: S7 Fig — Skin sections obtained from P5 pups were stained with toluidine blue. Increased infiltration of mast cells (black arrowheads) was found in Klk5-/-Sp5A135X/A135X mice. Scale bar, 100 μm. (TIF) [file pgen.1006566.s007.tif]

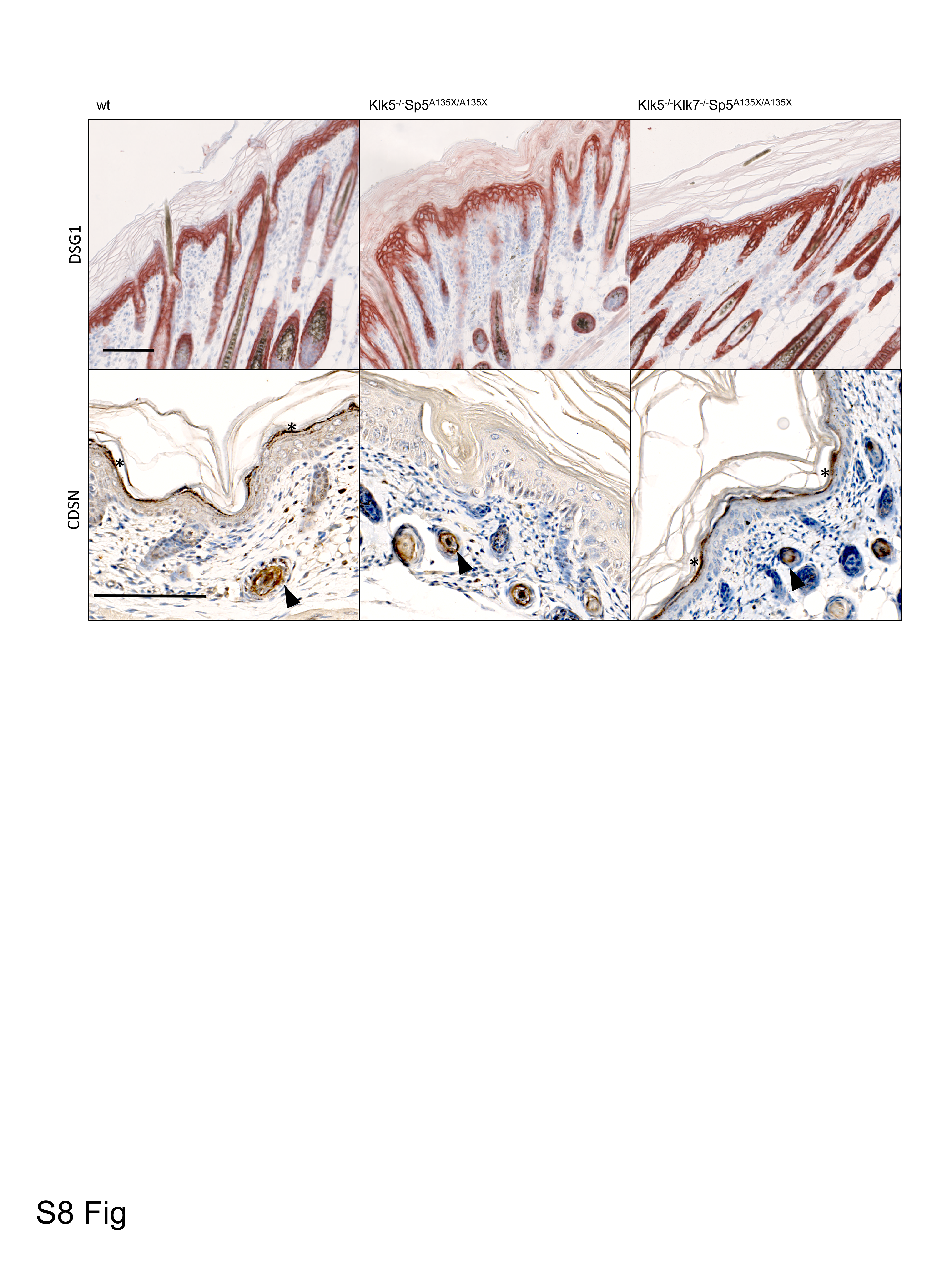

Supplement: S8 Fig — Skin sections obtained from P5 pups were stained with antibodies against DSG1 and CDSN. CDSN-positive staining was found in the inner root sheath (black arrowheads) and upper granular layer (asterisks) of wt and Klk5-/-Klk7-/-Sp5A135X/A135X. In contrast, only the inner root sheaths of non-hyperplastic hair follicles of Klk5-/-Sp5A135X/A135X P5 pups were CDSN-positive. No apparent staining was found at stratum granulosum/startum corneum interface or in the hyperkeratotic follicles. Scale bar, 100 μm. (TIF) [file pgen.1006566.s008.tif]

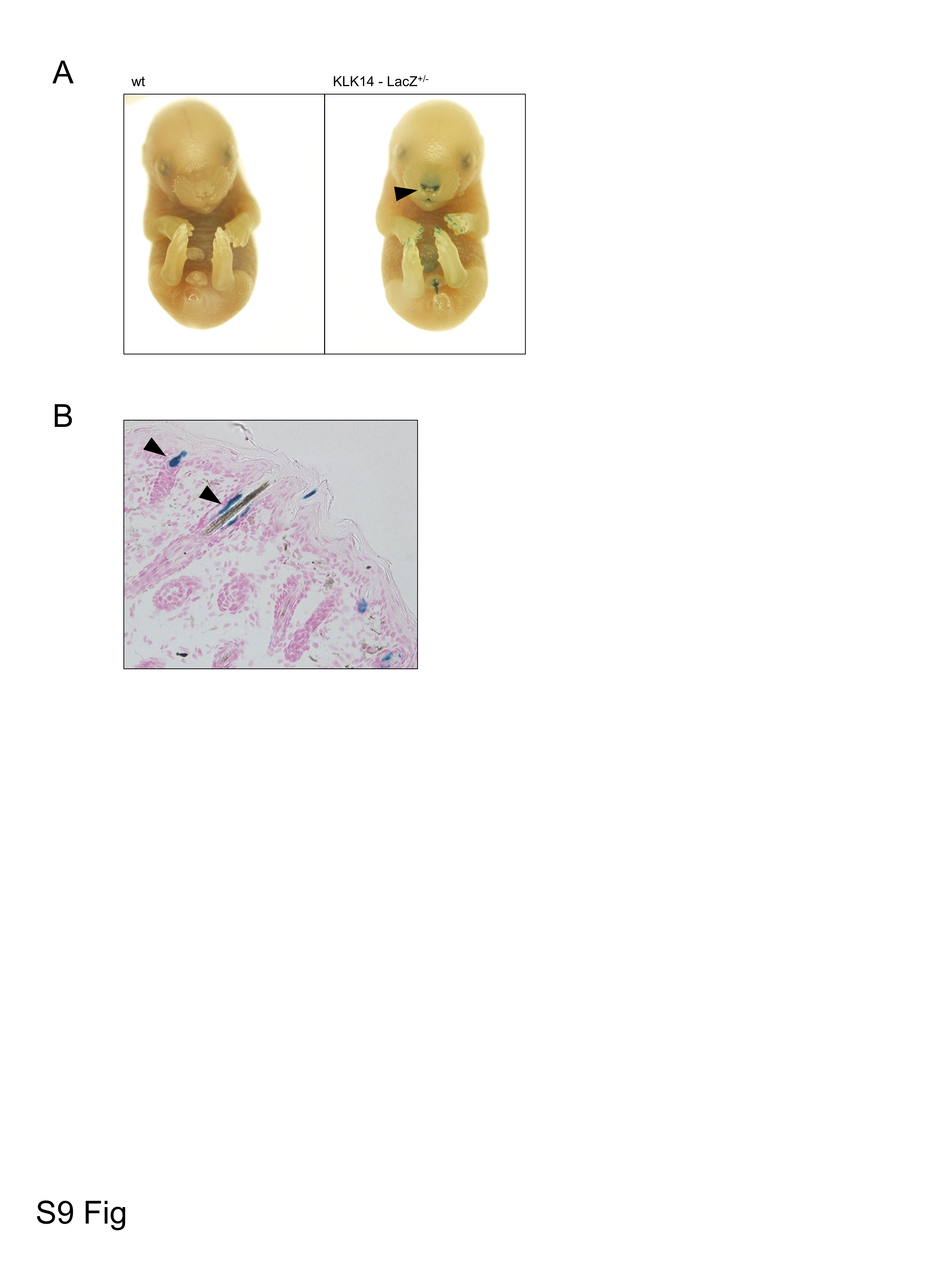

Supplement: S9 Fig — Klk14 expression was monitored using a mutant strain carrying LacZ reporter under the control of Klk14 promoter. (A) Analysis of Klk14 expression in 18.5 dpc embryos. Klk14 was found to be expressed in the area of nostrils (black arrowhead) (B) Analysis of P5 skin sections revealed specific expression of Klk14 in hair follicles. (TIF) [file pgen.1006566.s009.tif]
