# Supplementary material for: KLK5 and KLK7 Ablation Fully Rescues Lethality of Netherton Syndrome-Like Phenotype
Source: PLoS Genet. 2017 Jan 17;13(1):e1006566. doi: 10.1371/journal.pgen.1006566 (PMC5283769; doi:10.1371/journal.pgen.1006566)
Supplement: S1 Table — Sequences of primers used for qRT-PCR analysis. (DOCX) [file pgen.1006566.s010.docx]

**Supplementary Tables**

**Table S1. List of primers**

| **Primer designation** | **Primer Sequence (5´-3´)** |
| --- | --- |
| Spink5 qPCR F | ATGGGGATTTCATCTGTCCA |
| Spink5 qPCR R | AACATGGTTCTGGCTTTTCG |
| Klk5 qPCR F | CTGTTCTTGGTGGGGATGTT |
| Klk5 qPCR R | ATCCGTGCTGAGGTCTCTGT |
| Klk7 qPCR F | TGCAAAATGCGTCAGTACCA |
| Klk7 qPCR R | GACCTGGGTCTTTGTGGAGT |
| TSLP qPCR F | AGAAGCCCTCAATGACCA |
| TSLP qPCR R | TCTTGTGCCATTTCCTGAGTA |
| ICAM1 qPCR F | AACAGTTCACCTGCACGGAC |
| ICAM1 qPCR R | GTCACCGTTGTGATCCCTG |
| IL-1β qPCR F | AGTTGACGGACCCCAAAAGA |
| IL-1β qPCR R | GTGCTGCTGCGAGATTTGAA |
| IL-33 qPCR F | GCAGGAAAGTACAGCATTCAAGA |
| IL-33 qPCR R | GGGGAAATCTTGGAGTTGGAATAC |
